# Combined Effect of Pressure-Assisted Thermal Processing and Antioxidants on the Retention of Conjugated Linoleic Acid in Milk

**DOI:** 10.3390/foods4020065

**Published:** 2015-04-14

**Authors:** Sergio I. Martinez-Monteagudo, Marleny D.A. Saldaña

**Affiliations:** Department of Agricultural, Food and Nutritional Science, University of Alberta, Edmonton, AB T6G 2P5, Canada; E-Mail: marleny.saldana@ualberta.ca

**Keywords:** antioxidants, conjugated linoleic acid, dissolved oxygen, pressure-assisted thermal processing, phenolics

## Abstract

The effect of pressure-assisted thermal processing (PATP) in combination with seven synthetic antioxidants was evaluated on the retention of conjugated linoleic acid (CLA) in enriched milk. Milk rich in CLA was first saturated with oxygen, followed by the addition of either catechin, cysteine, ascorbic acid, tannic acid, gallic acid, caffeic acid or *p*-coumaric acid (500 mg kg^−1^ untreated milk). Samples were treated at 600 MPa and 120 °C up to 15 min of holding time. During PATP, CLA not only oxidized at a slower rate, but also less oxygen was consumed compared to the control (0.1 MPa and 120 °C). In addition, phenolic antioxidants were able to quench dissolved oxygen in samples treated with PATP. For those samples added with gallic acid and catechin, 85% and 75% of the CLA was retained after 15 min of holding time at 600 MPa and 120 °C, respectively. The retention of CLA was enhanced by the application of PATP in combination with gallic acid.

## 1. Introduction

Pressure-assisted thermal processing (PATP) has become a valuable alternative to traditional thermal treatments. Although sterilized products treated with PATP have not been commercialized yet, this technology has the potential to deliver a variety of novel products, and its further implementation at the industrial level is expected in the short to medium term [1].

There are three important characteristics of PATP. First, the rise in the sample temperature due to the heat of compression is used to reach the target or sterilization temperature, reducing the thermal damage that takes place in traditional sterilization processes [2]. Second, pressure reduces interatomic distance, affecting interactions, the bond energy which is distance dependent. Such interactions are van der Waals forces, electrostatic forces, hydrogen bonding and hydrophobic interactions of proteins. In contrast, covalent bonds are unlikely to be affected by pressure, because the bonding distance can be hardly further compressed [3]. This has been the main hypothesis in preserving the biological activity of functional compounds, such as ascorbic acid [4,5], folates [6,7], anthocyanins [8], lycopene [9] and conjugated linoleic acid [10]. Finally, the rate of a chemical reaction can be increased or decreased by pressure, according to whether the molar volume of the intermediate state (activated complex) is less or more voluminous. For a chemical reaction, the effect of pressure favours those reactions with a negative reaction volume and those reaction pathways with a negative activation volume [3].

Some authors have suggested that PATP can be used to produce superior quality products in cases where the traditional thermal treatments have failed to deliver high-quality products, such as egg-based and milk-based products, baby foods, desserts, gravies, soups and sauces [3,11].

In an effort to develop high-value milk products, various investigations have highlighted the health benefits of conjugated linoleic acid (CLA), a bioactive component naturally found in milk fat [12]. Unfortunately, CLA in milk suffers significant losses during heat treatment, including ultra-high temperature (UHT), as discussed elsewhere [13,14,15,16]. Interestingly, CLA was retained up to 90% when catechin, a potent antioxidant, was added at 1 g per kg of milk, regardless of the processing conditions used (60–120 °C and 100–600 MPa) [17]. These findings suggest that the use of antioxidants can enhance the retention of CLA in milk. However, their effectiveness and the way in which they capture free radicals or react with the dissolved oxygen to retard the free radical reactions have not been studied under PATP conditions. Understanding the antioxidant mechanisms under PATP conditions is important for product development. Thus, the objective of this study was to evaluate the combined effect of PATP and seven commonly-used antioxidants on the retention of CLA in milk.

## 2. Experimental Section

### 2.1 Obtaining CLA-Enriched Milk and Sample Preparation

Milk rich in CLA was obtained from the Dairy Research and Technology Centre at the University of Alberta (Edmonton, AB, Canada), following the protocol provided elsewhere [18]. Briefly, 12 lactating Holstein cows were first fed with a control diet for 3 days. Then, the cows were fed for 16 days with a diet supplemented with sunflower ground seed at 6% of dry matter. The diet consists of 60% forage and 40% concentrate. Cows were housed in tie stalls, and water was available at all times. The CLA-enriched milk was stored at −20 °C until needed. Prior to the PATP experiments, the CLA-enriched milk was thawed with running water at room temperature. Then, the milk rich in CLA was saturated with oxygen by bubbling oxygen for 45 min at room temperature. After that, five hundred milligrams of either catechin (CAT), cysteine (Cyst), ascorbic acid (AA), tannic acid (TAN), gallic acid (GA), caffeic acid (CAF) or *p*-coumaric acid (CUM) were added per one kg of untreated milk. All antioxidants used were reagent grade and were purchased from Sigma-Aldrich (Saint Louis, MO, USA). The antioxidants were dissolved using a bench homogenizer Diax 900 (Rose Scientific Ltd., Edmonton, AB, Canada).

### 2.2. Pressure-Assisted Thermal Processing

PATP experiments were carried out in 3-mL polypropylene tubes (Cryogenic vial, Fisher Scientific, Edmonton, AB, Canada). The tubes were filled with either raw CLA-enriched milk or CLA-enriched milk added with an antioxidant, capped and shaken before immersion in an oil bath at 102 °C. The preheating temperature was fixed, considering that the temperature of milk rises 3 °C per 100 MPa [19]. After 2 min, the pre-heated samples were transferred to the high pressure multivessel system (Apparatus U111 Unipress, Warszawa, Poland), where the vessels were already heated at 120 °C. The vessels were heated with a thermostat (Lauda Proline RP 855 Low Temperature, Lauda-Königshofen, Germany) using propylene glycol, which was also the pressure transmission fluid. The unit has four high pressure vessels with an internal volume of 8 mL. The temperature of the transmission fluid was recorded using a Type K thermocouple located at the bottom of each vessel. Then, the samples were pressurized at 600 MPa using a rate of 10 MPa s^−1^. Once 600 MPa was reached, the samples were held for 1, 5, 10 and 15 min. At the end of the holding time, the vessels were decompressed, and the samples were removed immediately from the high pressure vessels and cooled down with ice to avoid any further CLA degradation. For the control treatments, samples of raw CLA-milk in closed polypropylene tubes were pre-heated at 102 °C and subsequently transferred to an oil bath at 120 °C to imitate the PATP experimental run. For simplicity, the control treatments are referred to as samples treated at 0.1 MPa. PATP-treated and control samples were kept at −18 °C until further analysis. An additional set of experiments was conducted using different concentrations of CAT and GA (0, 100, 200, 300 and 500 mg kg^−1^). The samples were treated at 120 °C for 15 min at either 0.1 or 600 MPa. The CLA was expressed in terms of normalized (CLA_t_/CLA_o_) retained fraction from 0 to 1. The experimental protocol has been validated for kinetics studies elsewhere [10]. All experimental data were obtained in triplicate.

### 2.3. Analytical Determinations

#### 2.3.1. CLA Determination

The CLA content was determined by gas chromatography using a 100-m SP-2560 fused-silica capillary column, adopting the methodology previously described elsewhere [20]. The CLA content measured by the GC represented the total CLA.

#### 2.3.2. Dissolved Oxygen

The dissolved oxygen (DO_2_) in milk was measured using an oxygen meter (OM-4, Microelectrodes, Inc., Bedford, NH, USA). The oxygen meter was calibrated using a two-point calibration (0% and 100% of saturation). The electrode was immersed in 0% solution obtained by boiled and cooled water under nitrogen flushing. The 100% of saturation solution was obtained by bubbling oxygen for 45 min at room temperature in 500 mL of enriched milk. At each experimental point, the electrode was allowed to stabilize to the sample temperature before the DO_2_ measurement was recorded. The measurements of DO_2_ were performed in duplicate.

### 2.4. Data Analysis

#### 2.4.1. Retention of CLA

The retained CLA in the presence of oxygen during processing was described according to reaction as follows:

$$\text{CLA}+{\text{ O}}_{2}\overset{k_{1}}{\to }\text{ oxidation products}$$

where *k*_1_ is the oxidation rate constant (min^−1^) for each antioxidant, the *k*_1_ value was obtained by solving the following differential equation:

(1) $$\frac{d\text{CLA}}{dt}=\frac{d{\text{O}}_{2}}{dt}=-k_{1}\cdot \text{CLA}\cdot {\text{O}}_{2}$$

#### 2.4.2. Quenching Ability

An additional set of experiments was performed to determine the quenching ability of phenolic antioxidants in milk treated at 120 °C and 600 MPa. Quenching oxygen by phenolic antioxidants was described by a bimolecular mechanism that follows:

$${\text{O}}_{2}+\text{Q}\;\overset{k_{2}}{\to }\text{products}$$

where Q is the quenching agent or phenolic antioxidant; and *k*_2_ is the apparent quenching rate constant (min^−1^). Total phenolic content was considered as the quenching agent, and it was determined by the Folin-Ciocalteu method reported elsewhere [21]. Then, Equations (2)–(4) were solved simultaneously to obtain *k*_1_ and *k*_2_ values.

(2) $$\frac{d\text{CLA}}{dt}=-k_{1}\cdot \text{CLA}\cdot {\text{O}}_{2}$$

(3) $$\frac{d{\text{O}}_{2}}{dt}=-k_{1}\cdot \text{CLA}\cdot {\text{O}}_{2}-k_{2}\cdot {\text{O}}_{2}\cdot \text{Q}$$

(4) $$\frac{d\text{Q}}{dt}=-k_{2}\cdot {\text{O}}_{2}\cdot \text{Q}$$

The differential equations were solved using DYNAFIT (BioKin Ltd., Madison, WI, USA, www.biokin.com/dynafit).

## 3. Results and Discussion 

### 3.1. Pressure, Temperature and Time History

**Figure 1:**
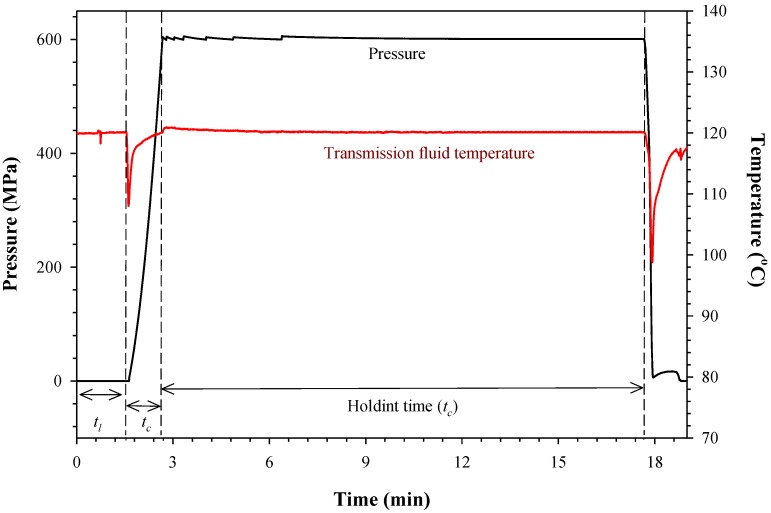
Typical pressure, temperature and time history during pressure-assisted thermal processing of conjugated linoleic acid (CLA)-enriched milk treated at 600 MPa/120 °C (*t*_l_, loading time; *t*_c_, compression time; *t*_h_, holding time).

Figure 1 shows the pressure and temperature history during PATP of CLA-enriched milk treated at 600 MPa and 120 °C for 15 min of holding time. The time needed to insert the preheated sample (102 °C), adjust the transmission fluid volume and close the high pressure vessel was considered as the loading time (*t*_l_). Likewise, the time required to reach isothermal and isobaric conditions was labelled as the compression time (*t*_c_). The temperature of the transmission fluid dropped 13 °C during compression, because the temperature of the pressurizing fluid that comes from the intensifier was lower (~75 °C) compared to the fluid temperature inside the vessel (120 °C). The temperature of the sample and the medium increased due to adiabatic heating, reaching a final temperature of 120 °C. The point where both isothermal and isobaric conditions have been reached was considered the start of the holding time (*t*_h_). It can be assumed that the temperature of the transmission fluid, sample and vessel are the same (120 °C) during the holding time. Knowledge of the temperature and pressure history during PATP is needed for a correct interpretation of the experimental findings. The reduction of CLA can be attributed to a combination of pressure, temperature and time rather than temperature itself. The processing conditions of 600 MPa and 120 °C for at least 5 min were enough to inactivate seven-log of *Bacillus amyloliquefaciens* in CLA-enriched milk and to inactivate 99.9% of alkaline phosphatase [22].

### 3.2. CLA-Enriched Milk

**Figure 2:**
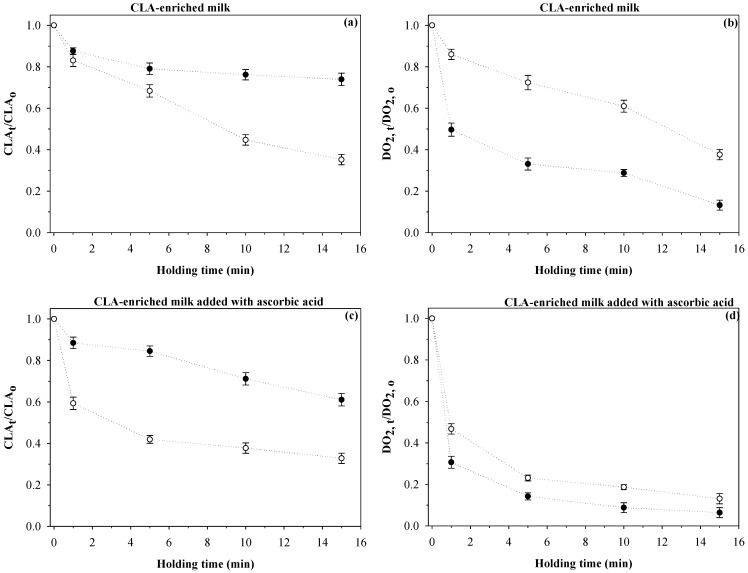
Changes in conjugated linoleic acid (CLA) and dissolved oxygen (DO_2_) in CLA-enriched milk treated at 120 °C and either 600 MPa (●) or 0.1 MPa (○).

Milk saturated with oxygen and treated at 120 °C for up to 15 min was used to induce oxidation and therefore to evaluate the role of different antioxidants. Figure 2a shows the CLA changes in CLA-enriched milk treated at 0.1 MPa/120 °C or 600 MPa/120 °C up to 15 min. At 0.1 MPa, the retained fractions of CLA were 0.44 (44%) and 0.35 (35%) after 10 and 15 min, respectively. Similarly, Martinez-Monteagudo and Saldaña [15] reported that 15%–21% of CLA was retained in CLA-enriched milk heated at 120 °C /0.1 MPa. The difference in the retained fractions can be attributed to the different batch milk samples used. In milk fortified with CLA (2% of CLA in the total fat), the retained CLA ranged from 79% to 95%, depending on the processing conditions (125–145 °C/2–20 s) [13,14]. The high temperature used (120 °C) induces oxidation of CLA via the formation of free radicals through thermolysis. In addition, the dissolved oxygen reacts with oxygen species to form oxygen radicals, such as hydroxyl (HO•), peroxyl (ROO•) and hydroperoxyl (HOO•). These radicals can easily act as initiators for the oxidation of CLA. This is in agreement with the changes in DO_2_ observed in Figure 2b. At 0.1 MPa, the remaining fractions of DO_2_ after 10 and 15 min were 0.61 and 0.37, respectively. At 600 MPa, CLA was remarkably stable compared to the control treatment at 0.1 MPa, retaining 0.76 and 0.74 after 10 and 15 min, respectively. Based on these results, pressure may influence the reaction mechanism by which CLA is oxidized. Experimental data from the literature showed that thermal decomposition through homolysis of single bond and two-bond scission is delayed by the application of pressure up to 395 MPa at 100 °C [23,24]. A deceleration in the decomposition or homolysis of unsaturated fatty acids might result in a protective effect of the CLA.

Figure 2b shows the normalized DO_2_ in CLA-enriched milk treated at PATP. The remaining fractions of DO_2_ at 600 MPa/120 °C were 0.28 and 0.13 after 10 and 15 min, respectively. Table 1 shows the *k_1_* values calculated from Equations (1) and (2). The *k_1_* value accounts for two contributions, CLA reduction and DO_2_ consumption, and its determination can be used to infer possible reaction mechanisms. The *k_1_* value for milk was lower at 600 MPa/120 °C compared with that at 0.1 MPa/120 °C (0.059 and 0.091 min^−1^, respectively), indicating that pressure had a protective effect. Similarly, Martinez-Monteagudo and Saldaña [10] showed that the combination of 600 MPa/120 °C induces isomerisation rather than oxidation of CLA in milk rich in CLA. Isomerization only changes the isomer distribution of CLA without changing the total amount of CLA. In this study, total CLA was quantified, but milk fat contains over 20 different CLA isomers for which *cis*-9/*trans*-11 is the predominant isomer (≥85%). There are two possible mechanisms for the isomerization of CLA, and the predominant mechanism depends on the oxygen content [25]. The first mechanism consists of the migration of the double bond, known as sigmatropic molecular rearrangements, which is induced by temperature in the absence of oxygen. The second mechanism occurs in the presence of oxygen through free radical chain reaction, similar to the initiation step of the autoxidation of linoleic acid (C18:2) [26]. Interestingly, the initiation and propagation steps of free radical polymerization are indeed accelerated by pressure [27]. Therefore, it is reasonable to hypothesize that isomerization of CLA through the free radical mechanism is the predominant reaction at 600 MPa/120 °C. Contrary to this, oxidation of CLA rather than isomerization is the predominant reaction at 0.1 MPa/120 °C.

**Table 1:** Oxidation rate constant (*k*_1_, min^−1^) of CLA-enriched milk treated at 120 °C and either 600 or 0.1 MPa.

Antioxidant	600 MPa		0.1 MPa	
	*k_1_*, min^−1^	95% CI	*k_1_*, min^−1^	95% CI
Milk	0.059	0.006	0.091	0.010
Ascorbic acid	0.081	0.009	0.366	0.050
Cysteine	0.030	0.005	0.194	0.051
Caffeic acid	0.041	0.015	0.078	0.020
Catechin	0.051	0.009	0.139	0.015
Gallic acid	0.092	0.018	0.150	0.036
*p*-Coumaric acid	0.042	0.011	0.118	0.011
Tannic acid	0.069	0.019	0.084	0.017

*k_1_* is obtained by solving Equations (1) and (2); 95% CI, 95% confidence interval.

### 3.3. Ascorbic Acid

Changes in CLA content in enriched milk added with AA and treated at sterilization conditions are shown in Figure 2c. The retained CLA contents after 10 and 15 min were 0.37 and 0.32 at 0.1 MPa/120 °C, respectively. After 15 min, the retained CLA was similar to that in CLA-enriched milk without antioxidant addition (~0.35; Figure 2a). The processing conditions of 120 °C and up to 15 min are rather severe and could oxidize the ascorbic acid, causing it to lose its antioxidant activity. In UHT milk, ascorbic acid is lost by up to 50%, depending on the severity of the treatment time (135–150 °C/4 s) [28]. On the other hand, the combination of AA and a pressure of 600 MPa at 120 °C enhanced the retention of CLA (0.61) after 15 min of treatment compared with the treatment at 0.1 MPa/120 °C (0.32) and milk without added antioxidant (0.35). One possible explanation is that pressure protects the AA, which further reacts with DO_2_ and free radicals. AA was used to inhibit the formation of aldehydes in milk treated at 655 MPa and 75 °C for 5 min [29]. An investigation on the retention of AA in pressurized phosphate buffer systems showed that AA remained unchanged at a moderate temperature of 50 °C and 850 MPa, while at a higher temperature of 80 °C and 850 MPa, the AA degradation became notorious. More importantly, AA can be used to enhance the stability of folates in pressurized orange and tomato juices [4]. Another investigation showed that the combination of 700 MPa and 110 °C accelerated the degradation rate of ascorbic acid up to 20 times in raspberries [5]. These variations can be attributed to differences in the food matrix, processing conditions and dissolved oxygen.

The changes in DO_2_ in milk with added AA are shown in Figure 2d. For those samples treated at 0.1 MPa, the DO_2_ rapidly decreased (0.23 remaining fraction) within 5 min of holding time. As the experiment proceeded, the remaining fractions of DO_2_ were 0.18 and 0.13 after 10 and 15 min, respectively. In the case of PATP, the retained fractions of DO_2_ were 0.08 and 0.06 after 10 and 15 min, respectively. A close examination of the *k_1_* values (Table 1) indicated that more oxygen was consumed by those samples with added AA and treated at 0.1 MPa/120 °C (0.366 min^−1^) compared with those samples with added AA and treated at 600 MPa/120 °C (0.081 min^−1^). In the presence of oxygen, the AA is converted to dehydroascorbic acid (DHA) by abstracting a proton, making it unstable [30]. At the beginning of the holding time, the AA might be acting as a scavenging agent due to its relatively high initial concentration (0.5 g kg^−1^) [31]. Moreover, the initial molar concentration of the AA ([AA]_o_ = 2.8 mM) is four-times higher than the initial molar concentration of oxygen ([DO_2_]_o_ = 0.68 mM). A molar ratio of at least 2 (AA/O_2_) was reported to protect other compounds from oxidation [4]. When most of the DO_2_ was consumed, the oxidation of CLA was evident. One possible reason is that the AA can be ionized when pressure is applied to form ascorbate, which is more reactive than the AA. Both compounds oxidize to produce hydrogen peroxide, which might trigger the late oxidation of CLA [32].

### 3.4. Cysteine

The remaining CLA in samples with added cysteine (Cyst) is shown in Table 2. At 0.1 MPa and 120 °C, the retained fractions of CLA were 0.59 and 0.56 after 10 and 15 min of holding time, respectively. An investigation showed that Cyst can be used to inhibit the formation of off-flavor compounds in UHT milk [29]. At 600 MPa, the retained fractions of CLA were 0.73 and 0.70 after 10 and 15 min, respectively. The combination of Cyst and pressure yielded a higher remaining fraction of CLA compared with the control treatment (0.1 MPa). Likewise, Cyst inhibited the formation of aldehydes and hydrogen sulfide in milk pressurized at 655 MPa and heated at 75 °C for 10 min [29].

On the other hand, the DO_2_ rapidly decreased with increasing holding time at 0.1 MPa, resulting in a value of 0.05 after 15 min of holding time. At 600 MPa, the DO_2_ fraction was considerable higher than that at 0.1 MPa with a value of 0.42 after 15 min of holding time. In the presence of oxygen, Cyst reacts with the hydroxyl radical to form sulfonic acid [30]. This could be the reason for the substantial reduction in the DO_2_ fraction observed at 0.1 MPa (Table 2). The *k*_1_ values reported in Table 1 at 600 and 0.1 MPa were 0.030 and 0.194 min^−1^, respectively, meaning that a small amount of oxygen was needed to oxidize CLA. Cyst has the ability to quench oxygen. An investigation on the reactivity of the O_2_ with amino acids showed that oxidation of amino acids was the predominant reaction rather than O_2_ quenching. Cyst possesses a thiol group, which is believed to act as an antioxidant by donating hydrogen to neutralize free radicals [33]. The ability of Cyst to quench free radicals depends on the state of protonation of the functional group [34]. An investigation on the oxidation of milk fat with different amino acids added showed that a non-protonated (NH_2_) amino group, such as Cyst, inhibited the oxidation [35]. In PATP experiments, the reaction system suffers a temporary and reversible modification in the pH, which might change the reaction mechanism [36].

**Table 2:** Changes in conjugated linoleic acid (CLA) and dissolved oxygen (DO_2_) in CLA-enriched milk added with either cysteine, caffeic acid or catechin and treated at 120 °C and 0.1 or 600 MPa.

Holding time (min)	Normalized retention of conjugated linoleic acid (CLA)								
	Cysteine		Caffeic acid				Catechin		
	0.1 MPa	600 MPa	0.1 MPa		600 MPa		0.1 MPa		600 MPa
0	1.00 ± 0.00a	1.00 ± 0.00a	1.00 ± 0.00a		1.00 ± 0.00a		1.00 ± 0.00a		1.00 ± 0.00a
1	0.73 ± 0.03b	0.81 ± 0.03b	0.91 ± 0.03a		0.74 ± 0.03b		0.95 ± 0.03b		0.87 ± 0.03b
5	0.63 ± 0.02c	0.72 ± 0.02c	0.79 ± 0.02b		0.71 ± 0.02c		0.77 ± 0.02c		0.81 ± 0.02c
10	0.59 ± 0.03d	0.73 ± 0.03cd	0.70 ± 0.02c		0.71 ± 0.03c		0.69 ± 0.02d		0.79 ± 0.03c
15	0.56 ± 0.02e	0.70 ± 0.03d	0.69 ± 0.02c		0.66 ± 0.03d		0.39 ± 0.02e		0.75 ± 0.03d
**Holding time (min)**	**Normalized dissolved oxygen (DO_2_)**								
	**Cysteine**		**Caffeic acid**			**Catechin**			
	**0.1 MPa**	**600 MPa**	**0.1 MPa**	**600 MPa**		**0.1 MPa**		**600 MPa**	
0	1.00 ± 0.00a	1.00 ± 0.00a	1.00 ± 0.00a	1.00 ± 0.00a		1.00 ± 0.00a		1.00 ± 0.00a	
1	0.13 ± 0.02b	0.79 ± 0.03b	0.88 ± 0.03b	0.78 ± 0.03b		0.78 ± 0.02b		0.71 ± 0.03b	
5	0.11 ± 0.03c	0.64 ± 0.01c	0.54 ± 0.01c	0.65 ± 0.01c		0.65 ± 0.03c		0.30 ± 0.04c	
10	0.11 ± 0.01c	0.61 ± 0.03d	0.29 ± 0.03d	0.56 ± 0.02d		0.56 ± 0.01d		0.14 ± 0.03d	
15	0.05 ± 0.02d	0.42 ± 0.04e	0.25 ± 0.02e	0.18 ± 0.03e		0.18 ± 0.02e		0.09 ± 0.03e	

Means ± standard deviation (*n* = 3); columns with different letters (a–f) are significantly different (*p* < 0.05).

### 3.5. Phenolic Antioxidants

Table 2 and Table 3 show the retention of CLA in milk added with different phenolic antioxidants. The highest retention fraction of CLA in milk was obtained after adding CAF (0.69), followed by TAN (0.64), CUM (0.43), CAT (0.39) and GA (0.36) after 15 min of holding time at 120 °C/0.1 MPa. On the other hand, after 15 min and 120 °C/600 MPa, the highest retention fraction was obtained with GA (0.85), followed by CAT (0.75), CAF (0.66) CUM (0.63) and TAN (0.62). In general, phenolic antioxidants possess the ability to inhibit the autoxidation of lipids by trapping intermediate radicals and quenching dissolved oxygen due to their low ionization potential [37]. Table 2 and Table 3 also show the changes in DO_2_ in milk with added phenolic antioxidants. After 15 min of holding time at 120 °C/0.1 MPa, the consumption of DO_2_ was larger in those samples with added TAN and GA (0.11), followed by CAT (0.18), CUM (0.21) and CAF (0.25). After 15 min of holding time at 120 °C/600 MPa, the consumption of DO_2_ was greater in samples with added CUM (0.10), CAT (0.11) and GA (0.11), followed by TAN (0.13) and CAF (0.18). Upon thermal treatment, TAN is hydrolyzed and formed gallic acid and polygalloyls, which enhanced the antioxidant capacity. An investigation on the oxidation of soybean oil showed that the addition of hydrolyzed TAN extended the oxidation induction period by up to 84% compared with the control treatment [35]. These authors thermally treated TAN (121 °C up to 60 min) and found that 15 min of thermal treatment was enough to hydrolyze TAN. The ability of TAN to react with oxygen was demonstrated during the oxidation of ascorbate in the presence of copper, where TAN not only reacted with oxygen, but also chelated metal ions, delaying the oxidation process [38]. In PATP-treated samples, the *k*_1_ value was 0.069 min^−1^, suggesting a change in the reaction mechanism induced by pressure in the presence of TAN (Table 1).

The value of *k*_1_ (Table 1) in milk with CAF addition obtained at PATP was 0.041 min^−1^, indicating that the addition of CAF enhanced the retention of CLA. In the case of samples added with CAT, the *k*_1_ values were 0.051 and 0.139 min^−1^ at 600 and 0.1 MPa, respectively (Table 1). The *k*_1_ values indicated that a significant amount of oxygen was needed to oxidize CLA. For those samples added with GA, the *k*_1_ values were 0.092 and 0.150 min^−1^ at 600 and 0.1 MPa, respectively. Finally, those samples added with CUM yielded *k*_1_ values of 0.042 and 0.118 min^−1^ at 600 and 0.1 MPa, respectively. During pressure treatment, free phenols are probably ionized, and therefore, their ability to quench oxygen is significantly enhanced. Phenols are susceptible to ionization by pressure due to charge delocalization between the oxygen and aromatic ring, yielding molar activation volumes between −8 and −20 cm^3^ mol^−1^ [37]. Phenols form hydrogen bonds with surrounding molecules. Free phenols, on the other hand, are likely to be ionized, and the donated proton rapidly neutralizes the free radicals. In PATP, the use of phenolic antioxidants not only enhances the retention of CLA, but also quenches oxygen, which avoids isomerization, therefore protecting the biological activity of CLA.

**Table 3:** Changes in conjugated linoleic acid (CLA) and dissolved oxygen (DO_2_) in CLA-enriched milk added with either gallic acid, *p*-coumaric acid or tannic acid and treated at 120 °C and 0.1 or 600 MPa.

Holding time (min)	Normalized retention of conjugated linoleic acid (CLA)					
	Gallic acid		*p*-Coumaric acid		Tannic acid	
	0.1 MPa	600 MPa	0.1 MPa	600 MPa	0.1 MPa	600 MPa
0	1.00 ± 0.00a	1.00 ± 0.00a	1.00 ± 0.00a	1.00 ± 0.00a	1.00 ± 0.00a	1.00 ± 0.00a
1	0.69 ± 0.03b	0.98 ± 0.02b	0.91 ± 0.03b	0.87 ± 0.03b	0.91 ± 0.03b	0.78 ± 0.03b
5	0.61 ± 0.02c	0.94 ± 0.02c	0.76 ± 0.04c	0.75 ± 0.02c	0.78 ± 0.02c	0.76 ± 0.02c
10	0.51 ± 0.02d	0.89 ± 0.03d	0.53 ± 0.02d	0.72 ± 0.03d	0.75 ± 0.02d	0.72 ± 0.03d
15	0.36 ± 0.02e	0.85 ± 0.03e	0.43 ± 0.03e	0.63 ± 0.03e	0.64 ± 0.02e	0.62 ± 0.03e
**Holding time (min)**	**Normalized dissolved oxygen (DO_2_)**					
	**Gallic acid**		***ρ*-Coumaric acid**		**Tannic acid**	
	**0.1 MPa**	**600 MPa**	**0.1 MPa**	**600 MPa**	**0.1 MPa**	**600 MPa**
0	1.00 ± 0.00a	1.00 ± 0.00a	1.00 ± 0.00a	1.00 ± 0.00a	1.00 ± 0.00a	1.00 ± 0.00a
1	0.18 ± 0.03b	0.68 ± 0.02b	0.84 ± 0.02b	0.47 ± 0.03b	0.63 ± 0.03b	0.27 ± 0.03b
5	0.17 ± 0.02b	0.41 ± 0.04c	0.27 ± 0.04c	0.18 ± 0.01c	0.33 ± 0.02c	0.21 ± 0.01c
10	0.13 ± 0.02c	0.12 ± 0.03d	0.23 ± 0.03d	0.11 ± 0.02d	0.30 ± 0.03d	0.19 ± 0.02d
15	0.11 ± 0.02c	0.11 ± 0.03d	0.21 ± 0.02e	0.10 ± 0.02d	0.11 ± 0.02e	0.13 ± 0.02e

Means ± standard deviation (*n* = 3); columns with different letter (a–f) are significantly different (*p* < 0.05).

### 3.6. Different Concentrations of Catechin and Gallic Acid

**Figure 3:**
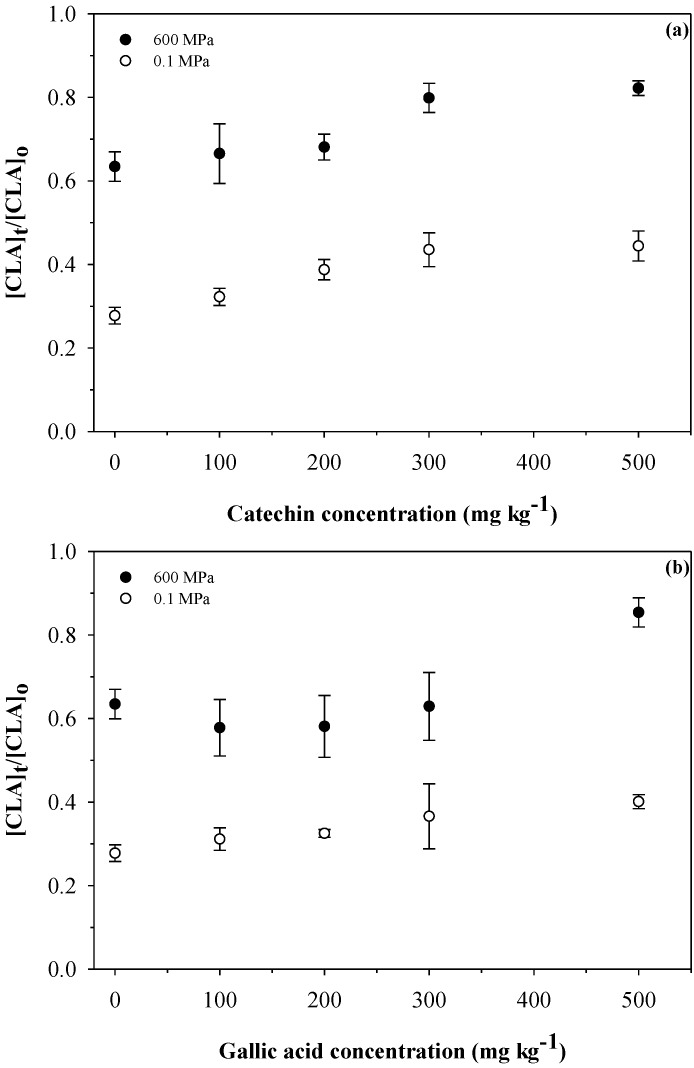
Effect of the concentration of (**a**) catechin and (**b**) gallic acid on the CLA retention in CLA-enriched milk treated at 120 °C for 15 min at either 0.1 or 600 MPa.

An additional set of experiments were conducted using different concentrations of CAT and GA (0, 100, 200, 300 and 500 mg kg^–1^, Figure 3). The samples were treated at 120 °C for 15 min at either 0.1 or 600 MPa. Using catechin and gallic acid, better CLA retention was obtained at 600 MPa than at 0.1 MPa. In the case of milk with added CAT and treated at 0.1 MPa (Figure 3a), the retention of CLA was enhanced from 0.27 to 0.44 as the concentration increased up to 500 mg kg^–1^. At 600 MPa, the retention of CLA was remarkably enhanced, reaching values of 0.82 when 500 mg kg^–1^ of CAT was added. Similarly, the combined effect of 600 MPa and GA resulted in a retention of 0.85, which is considerably higher than that obtained at 0.1 MPa (0.41). Catechin has been used for delaying non-enzymatic browning in UHT milk [39]. It was demonstrated that a concentration of 0.1 mmol L^–1^ was enough to inhibit the browning of UHT non-enriched milk at 145 °C for 15 s during prolonged storage up to 36 days. More importantly, consumer sensory analysis showed no difference with respect to the control samples (samples without added catechin) [39]. In our study, a similar concentration of CAT was used (500 mg kg^−1^, 0.17 mmol L^−1^); therefore, changes in sensory perception due to the addition of CAT are not expected, but further research is needed to confirm this assumption.

### 3.7. Possible Reaction Mechanisms of Phenolic Antioxidants

The use of phenolic antioxidants (500 mg kg^−1^) in combination with high pressure yielded a high retention of CLA (~85% in the case of GA), even at a prolonged holding time of 15 min. In general, the effectiveness of a given antioxidant is usually associated with the structure-function relation, in which the number of –OH groups are related to its effectiveness. In the case of the retention of CLA upon pressure, the number of –OH groups did not reflect the antioxidant effectiveness. While TAN possess 27 –OH groups in its structure, the retained normalized CLA was only 0.633. In contrast, GA and CAT with four and five –OH groups, respectively, yielded higher retention values (>0.75) than that obtained using TAN. The stability of antioxidants played an important role in enhancing the retention of CLA. The GA is considered a stable molecule, as proven by its thermogravimetric analysis, which showed that decarboxylation and further degradation started only at 260 °C [40]. A closer observation of Table 2 and Table 3 revealed that the oxygen was gradually consumed while the CLA remained unchanged. Phenolic antioxidants are known for their ability to quench oxygen, preventing the initiation stage of autoxidation [34]. Table 4 shows the changes in total phenolic content for milk samples with added 500 mg kg^−1^ of GA, CAT and CAF and treated at 120 °C/600 MPa.

**Table 4:** Normalized phenolic content in CLA-enriched milk added with different phenolic antioxidants and treated at 120 °C and 600 MPa.

Holding time (min)	Antioxidant added (500 mg kg^−1^)		
	Gallic acid	Catechin	Caffeic acid
0	1.00 ± 0.00	1.00 ± 0.00	1.00 ± 0.00
1	0.94 ± 0.02	0.72 ± 0.03	0.94 ± 0.02
5	0.81 ± 0.01	0.53 ± 0.04	0.81 ± 0.01
10	0.61 ± 0.02	0.44 ± 0.05	0.61 ± 0.04
15	0.48 ± 0.07	0.40 ± 0.02	0.48 ± 0.05
*k*_1_ (min^−1^)	0.029 ± 0.006	0.078 ± 0.005	0.014 ± 0.005
*k*_2_ (min^−1^)	0.081 ± 0.006	0.051 ± 0.019	0.061 ± 0.025

The variability of *k*_1_ and *k*_2_ correspond to the 95% confidence interval.

In general, the phenolic content followed a similar pattern to that observed for the DO_2_, reaching normalized retention values in the range of 0.40–0.48 after 15 min of holding time. Table 4 also shows the apparent quenching rate constant. Interestingly, GA yielded the highest *k*_2_ value (quenching constant rate) and the lowest *k*_1_ value (oxidation constant rate of CLA) followed by CAT and CAF at 120 °C/600 MPa up to 15 min. The use of GA resulted in the highest CLA retention followed by CAT. It seems that GA has the ability to quench oxygen and, therefore, to delay the oxidation of CLA. Another important factor to consider is the ratio of DO_2_ to the antioxidant. Ratios for GA and CAT were calculated, as these antioxidants yielded the highest retention of CLA. After 10 min of holding time, ratios of 0.22 and 1.28 were obtained for GA and CAT, respectively. A ratio of DO_2_/antioxidant higher than one means that the antioxidant is consumed faster than the available oxygen, indicating that there is an excess of oxygen that can further react. On the other hand, a DO_2_/antioxidant ratio of less than one could be viewed as the amount of oxygen being limited with respect to the amount of antioxidant (GA). These calculated ratios further demonstrated that the use of GA significantly enhanced the retention of CLA during PATP of milk. The processing conditions of temperature, pressure and holding time not only influenced the retention of CLA, but also the effectiveness of the tested phenolic antioxidants. Further studies should determine the content of each antioxidant used after PATP treatments to evaluate its stability in the enriched milk. 

## 4. Conclusions

In the presence of oxygen, CLA was remarkably stable in samples treated with PATP compared to the control. The analysis of remaining CLA and consumed oxygen suggests that pressure-induced isomerization of CLA, which might occur through a free radical mechanism. The application of PATP in milk samples containing phenolic antioxidants enhanced the retention of CLA. Gallic acid yielded the highest retention of CLA followed by catechin. During PATP, the rate of oxygen quenching was higher than the oxidation rate of CLA, which might avoid isomerization and, therefore, preserve the biological activity of CLA. Thus, the combination of PATP and the use of phenolic antioxidants can be applied to produce milk-based beverages rich in CLA, addressing the growing demand for functional drinks.
